# Supplementary material for: Modeling glioblastoma heterogeneity as a dynamic network of cell states
Source: Mol Syst Biol. 2021 Sep 16;17(9):e10105. doi: 10.15252/msb.202010105 (PMC8444284; doi:10.15252/msb.202010105)
Supplement: Supplementary file 6 — Source Data for Figure 5 [file MSB-17-e10105-s004.zip › Figure5A_sourcedata/GSEA_3017/hallmarks_stateC.GseaPreranked.1621934520804/HALLMARK_MYOGENESIS.html]

Details for gene set HALLMARK\_MYOGENESIS[GSEA]

|  || Dataset | state13017 |
| Phenotype | NoPhenotypeAvailable |
| Upregulated in class | na\_neg |
| GeneSet | HALLMARK\_MYOGENESIS |
| Enrichment Score (ES) | -0.386222 |
| Normalized Enrichment Score (NES) | -1.5684032 |
| Nominal p-value | 0.05448718 |
| FDR q-value | 0.07323608 |
| FWER p-Value | 0.464 |
Table: GSEA Results Summary

  

Fig 1: Enrichment plot: HALLMARK\_MYOGENESIS      
 Profile of the Running ES Score & Positions of GeneSet Members on the Rank Ordered List

  

| PROBE | GENE SYMBOL | GENE\_TITLE | RANK IN GENE LIST | RANK METRIC SCORE | RUNNING ES | CORE ENRICHMENT || 1 | CKB |  |  | 102 | 0.335 | -0.1243 | No |
| 2 | PKIA |  |  | 129 | 0.309 | -0.1215 | No |
| 3 | APOD |  |  | 222 | -0.259 | -0.2403 | No |
| 4 | IGFBP7 |  |  | 225 | -0.259 | -0.2039 | No |
| 5 | DTNA |  |  | 281 | -0.271 | -0.2569 | No |
| 6 | ABLIM1 |  |  | 348 | -0.297 | -0.3250 | No |
| 7 | COL4A2 |  |  | 353 | -0.300 | -0.2857 | No |
| 8 | ITGB5 |  |  | 356 | -0.300 | -0.2429 | No |
| 9 | WWTR1 |  |  | 437 | -0.345 | -0.3276 | No |
| 10 | ADAM12 |  |  | 472 | -0.374 | -0.3286 | Yes |
| 11 | NQO1 |  |  | 479 | -0.381 | -0.2801 | Yes |
| 12 | HBEGF |  |  | 480 | -0.386 | -0.2206 | Yes |
| 13 | SCD |  |  | 504 | -0.431 | -0.1938 | Yes |
| 14 | CRYAB |  |  | 536 | -0.485 | -0.1726 | Yes |
| 15 | IGFBP3 |  |  | 538 | -0.493 | -0.0983 | Yes |
| 16 | SPARC |  |  | 567 | -0.575 | -0.0580 | Yes |
| 17 | CLU |  |  | 581 | -0.690 | 0.0259 | Yes |
Table: GSEA details [plain text format]

  

Fig 2: HALLMARK\_MYOGENESIS: Random ES distribution      
 Gene set null distribution of ES for **HALLMARK\_MYOGENESIS**
